# Tailoring Flame Retardance and Thermal Conductivity of Epoxy/Benzoxazine Mixtures via Aluminum Trihydrate and Ceramic Hybridization

**DOI:** 10.3390/polym18050648

**Published:** 2026-03-06

**Authors:** Kyung-Soo Sung, Hye-Won Cho, Kyu-Hwan Kwon, Namil Kim

**Affiliations:** 1 Department of Chemical Engineering, Hannam University, Daejeon 34054, Republic of Korea; kssung@protavic.co.kr (K.-S.S.); khkwon2100@naver.com (K.-H.K.); 2 Korea Research & Development Center, Protavic Korea, Daejeon 34326, Republic of Korea; hwcho@protavic.co.kr

**Keywords:** aluminum trihydrate, benzoxazine, composites, flame retardancy, thermal conductivity

## Abstract

A composite meeting the UL94 V-0 rating was produced by adding 30 wt% epoxy silane-modified aluminum trihydrate (EPATH) to a 60/40 epoxy/benzoxazine matrix. Various bimodal and trimodal composites containing 20–40 wt% of three types of ceramic fillers, i.e., aluminum oxide (Al_2_O_3_), boron nitride (BN), and magnesium oxide (MgO), were prepared to simultaneously achieve flame-retardant and thermal conductive properties. The bimodal composites with 40 wt% of Al_2_O_3_ and MgO exhibited thermal conductivities of 1.22 W/m∙K and 1.29 W/m∙K, respectively, which were superior to that of the composite containing the same amount of ATH (1.0 W/m∙K). In contrast, both the coefficient of thermal expansion (CTE) and shear strength decreased with increasing ceramic filler content. For agglomerated BN, the filler loading was constrained above 30 wt% because its high specific volume caused a significant rise in the viscosity. In the trimodal composites with a total filler content of 40 wt% of Al_2_O_3_ and BN, a BN fraction of 7.5 wt% yielded the highest thermal conductivity of 1.64 W/m∙K and the lowest water absorption of 0.69%. When the trimodal composites were exposed to −55 °C and 150 °C for 1000 h, they exhibited a reduction in shear strength of less than 30% compared to their initial values.

## 1. Introduction

Power modules enable energy conversion, high-speed switching, and system management in electric vehicles, renewable energy systems, and high-density data centers. One of the critical issues faced is heat management, as significant heat generation can affect reliability, performance, and safety. Power semiconductor dies, such as silicon carbide (SiC) and gallium nitride (GaN), are increasingly operated at temperatures exceeding 200 °C due to their high-power densities [1,2,3,4]. Given that their performance is highly sensitive to temperature fluctuation, increasingly stringent requirements have been established for both the thermal conductivity and long-term thermal stability of packaging materials. Encapsulants used to protect the semiconductor chips and wire bond from environmental and mechanical damage play several critical roles to achieve successful operation and reliability of power electronics. They must possess a specific set of electrical, thermal, and mechanical properties to ensure the reliability and longevity of electrical components. For instance, a mismatch of coefficient of thermal expansion (CTE) between the encapsulants and the electrical components leads to device failure at high temperatures [5,6]. The thermal stress may be suppressed by lowering the thermal expansion mismatch between the matrix and the die. For the same weight percentage, smaller particles reduce the CTE more effectively than larger microparticles. Therefore, the criteria for choosing proper encapsulants are challenging because they should possess a combination of physical, chemical, and mechanical properties that are suitable for safe and reliable use in operating conditions.

The encapsulants for power module systems must satisfy the stringent requirements for thermal transport and stability, along with effective flame retardancy to prevent heat-induced mechanical failure and fire hazards [7,8,9,10]. The choice of soft silicone gels or hard epoxies is determined by the specific thermomechanical properties and operating environment. Most base polymeric resins possess inherent thermal insulation properties and high flammability, necessitating the use of additives to meet the rigorous safety and reliability standards. Thermal conductivities of conventional epoxy molding compounds (EMCs), typically comprising an epoxy matrix and fused silica (SiO_2_), and silicone gel (potting) are reported to be approximately 0.8–1.0 W/m·K and 0.15–0.2 W/m∙K, respectively [11]. A simple and efficient strategy for enhancing thermal conductivity involves the incorporation of thermally conductive ceramic fillers such as aluminum oxide (Al_2_O_3_), boron nitride (BN), and magnesium oxide (MgO), while maintaining electrical insulation [12,13,14]. The degree of thermal improvement varies based on the intrinsic properties, size, and shape of the specific ceramic species. The widespread application of epoxy resins in the electronic packaging is often constrained by high flammability due to the combustible nature of the hydrocarbon structure. Aluminum trihydrate (ATH) is a prevalent flame retardant for polymeric resins due to its cost-effectiveness and the evolution of non-toxic gaseous byproducts [15,16,17]. However, its application is limited by the high filler loadings, often exceeding 60 wt%, which inevitably leads to deterioration of mechanical properties and flow-related defects during the molding due to significantly increased viscosity. Benzoxazine resins are widely employed in industrial applications, such as coating, adhesives, and encapsulants, due to their outstanding dimensional stability derived from low cure shrinkage and high flame resistance [18,19,20]. However, these resins suffer from high cure temperature and low crosslinking density caused by intermolecular hydrogen bonding. To overcome the drawbacks of epoxy resin, benzoxazine is often copolymerized. In our previous study, we focused on providing thermal resistance and flame-retardant characteristics to the matrix via epoxy–benzoxazine copolymerization. A 60/40 benzoxazine/epoxy blend was determined to be the optimal composition following a systematic evaluation of various mixing ratios [21]. The mixture revealed good thermomechanical properties and satisfied a UL-94 V0 rating at only 30 wt% ATH. This implies that the typical issues of reduced mechanical properties and poor processability associated with high ATH loading can be mitigated. Furthermore, the introduction of a silane coupling agent on the ATH surface improved its dispersibility and reduced the viscosity. However, the thermal conductivity of the composites remains unsatisfactory for high-power electronics, exhibiting below 1.0 W/m∙K even at 60 wt% ATH loading.

In the present study, three distinct ceramic fillers, including aluminum oxide (Al_2_O_3_), boron nitride (BN), and magnesium oxide (MgO), were further added to a flame-retardant composite comprising a 60/40 epoxy/benzoxazine (Epoxy/BZ) blend and 30 wt% surface-modified ATH (EPATH) to supplement the thermal conductivity limitations of ATH. ATH with a similar particle size was also incorporated at the same load levels of ceramic fillers for comparison. Various bimodal and trimodal composites were fabricated by varying the filler composition and loading. Their properties, such as viscosity, coefficient of thermal expansion (CTE), viscoelastic properties, water absorption, shear strength, and thermal conductivity, were systematically compared to identify the optimal formulation that balances flame retardancy and thermal conductivity.

## 2. Materials and Methods

### 2.1. Materials and Sample Preparation

A blend of cycloaliphatic epoxy (CELLOXIDE 2021P, Daicel Chemical Industries, Tokyo, Japan) and benzoxazine (P-d type, Shikoku Chemicals, Kagawa, Japan) was prepared at a weight ratio of 60/40. The mixture was copolymerized with the addition of 50 wt% curing agent (RIKACID MH-T, New Japan Chemical, Osaka, Japan) and 5 wt% accelerator (2E4MZ-CN, Shikoku Chemicals, Kagawa, Japan). Aluminum trihydrate (SG25 LSA, Sibelco, Antwerpen, Belgium) with a mean particle size of 5.0 μm was incorporated into 60/40 epoxy/benzoxazine blend at a fixed loading of 30 wt%, after being surface-modified with an epoxy-containing silane coupling agent (KBM403, ShinEtsu Chemicals, Tokyo, Japan). For surface modification, 1.0 g of coupling agent was dissolved in 200 mL of ethanol, and then 100 g of ATH was slowly added to the solution. The mixtures were homogenized via mechanical stirring at room temperature and heated up to 60 °C for the reaction. The product was filtered and washed with deionized water several times. Silane-treated ATH was dried at 90 °C in a convection oven for 12 h [21]. Three different ceramic fillers were added as secondary reinforcement to enhance the thermal conductivity of epoxy/benzoxazine/EPATH composites. Spherical-shaped aluminum oxide (Al_2_O_3_, mean size: 75 µm) and magnesium oxide (MgO, mean size: 90 µm) were acquired from Denka Corp. Ltd. (Tokyo, Japan). Agglomerated boron nitride (BN) with a mean particle size of 130 µm was obtained from 3M Co., Ltd. (Kempten, Germany). For comparison, ATH (H-WF-100, Zibo Joyreach New Materials Co., Ltd., Zibo, China) with a mean particle size of 100 µm was selected and incorporated at the same loading levels as the ceramic fillers, and their properties were evaluated.

Each filler was uniformly dispersed within the epoxy/benzoxazine resin using a three-roll mill (EXAKT 80E, EXAKT, Norderstedt, Germany). The mixtures were then degassed in a vacuum bath at 100 rpm for 30 min to eliminate entrapped air. The prepared mixtures were stored in a freezer at −40 °C prior to use. The curing process was carried out at 175 °C for 60 min. Isothermal DSC analysis at 175 °C revealed that while the exothermic peak intensity was dependent on the filler loading, the curing process was finalized within 10 min for all compositions (Figure S1). The compositions of the bimodal and trimodal epoxy/benzoxazine composites are listed in Table 1. In all formulations, the EPATH content was fixed at 30 wt%, whereas the content of additional ATH or ceramic fillers varied from 20 to 40 wt%. Composites containing 40 wt% BN could not be fabricated due to excessively high viscosity.

### 2.2. Characterization

The UL94 vertical flammability test for the epoxy/benzoxazine/EPATH composites was conducted using specimens with dimensions of 127 mm in length, 12.7 mm in width, and 3.2 mm in thickness. The viscosity of the bimodal and trimodal epoxy/benzoxazine mixture was measured using a Brookfield rotational viscometer (DV2T, Brookfield Engineering Labs, Middleboro, MA, USA) at room temperature by varying the spindle rotation speed from 0.2 rpm to 2 rpm. The adhesion property was investigated through a die shear test (Dage Series 4000, Aylesbury, UK). Adhesive paste was applied on a silver (Ag)-plated lead frame, and then a square-shaped silicon (Si) die with a dimension of 1.25 mm in length and 350 µm in thickness was attached. Following thermal curing, the shear force required to detach the Si die from the Ag lead frame was determined at a crosshead speed of 100 µm/s. The data presented are the average of five independent tests.

The coefficient of thermal expansion (CTE) of the composites was determined by measuring the dimensional change as a function of temperature using a thermomechanical analyzer (TMA, Model 2940, Eden Prairie, MN, USA). The samples were heated from 20 °C to 250 °C at a heating rate of 5 °C/min. The viscoelastic properties were evaluated in tension mode using a dynamic mechanical analyzer (Pyris Diamond DMA, Perkin Elmer, Waltham, MA, USA). The cured specimens (200 mm in length × 6 mm in width × 0.3 mm in thickness) were heated from 0 °C to 280 °C at a heating rate of 2 °C/min under a nitrogen atmosphere. The dynamic mechanical measurements were conducted at a fixed frequency of 1 Hz. Thermal conductivity measurement was carried out using the laser flash method (LFA447, Netzsch Instruments, Selb, Germany). Thermal conductivity was calculated from the thermal diffusivity, which was measured using square sheet specimens (10 mm in length and 1 mm in thickness). The data presented are the average of three independent tests. The crosslinking density ($\nu_{c}$) of the composites was calculated from the following equation [22]:

$$\nu_{c}=\frac{E_{e}^{\prime }}{3RT}$$

where *R* is the gas constant, *T* is the absolute temperature at *T*_g_ + 50 °C, and $E_{e}^{\prime }$ is the equilibrium storage modulus at *T*_g_ + 50 °C.

The water absorption of the trimodal composites was evaluated via the gravimetric method. The entire specimens were immersed in water at room temperature for 24 h. Upon removal, surface moisture was carefully wiped off, and the subsequent mass change was recorded to calculate water absorption. The mean water absorption value was calculated from five independent specimens. Thermal aging was conducted at −55 °C and 150 °C for a total duration of 1000 h. The specimens, Si die bonded to an Ag-plated lead frame, were stored in a freezer (DF9017, IlShinBioBase Co. Ltd., Dongducheon, Republic of Korea) and a convection oven (OF-02, Jeio Tech, Daejeon, Republic of Korea) maintained at their respective temperatures. Following the thermal aging, the specimens were removed from the chamber and stabilized at room temperature for 1 h prior to the measurements. The variation in shear strength was monitored every 200 h.

## 3. Results

### 3.1. Thermal and Mechanical Properties of Bimodal Epoxy/Benzoxazine Composites

Viscosity is a critical determinant of processability in microelectronic packaging, primarily influenced by the composition of the epoxy/benzoxazine (epoxy/BZ) matrix and the filler content. While high-temperature viscosity is typically characterized for large-scale applications, room-temperature viscosity is more commonly utilized for small-scale or one-component systems. Accordingly, the viscosity in this study was measured at ambient temperature. For syringe-dispensed encapsulants, the viscosity at 2–5 rpm represents the discharge flow, whereas the viscosity at 0.2–0.5 rpm indicates the resistance to flow when stopped. Dispensing occurs at an elevated temperature for bulk application or a two-component system. However, one-component systems or small-scale applications typically utilize viscosity values measured at ambient temperature. Viscosity measurements at elevated temperatures are not conducted in this study to evaluate the workability of the paste at ambient temperature. Table 2 summarizes the viscosity at 0.2 and 2 rpm across the composite systems. It is noticed that all samples exhibit shear thinning behavior, characterized by a decrease in viscosity with increasing shear rate. For instance, the viscosity of the Bi-EPATH/ATH series ranges from 74,560 cPs to 194,700 cPs at 0.2 rpm and 14,290 cps to 119,300 cps at 2 rpm. Similar trends are observed across the other bimodal systems. The incorporation of BN led to a dramatic surge in viscosity, exceeding the instrument measurement limit at loadings above 30 wt%. This phenomenon is directly attributed to the exceptionally low bulk density of BN (0.5 g/cm^3^). At equivalent weight fractions, BN occupies a larger volume compared to high-density ceramics such as Al_2_O_3_ (~3.9 g/cm^3^), MgO (~3.5 g/cm^3^), or ATH (~2.4 g/cm^3^). Because flow resistance is fundamentally governed by the volume fraction of dispersed solid particles, the large spatial volume of BN hinders flow, thereby increasing the viscosity. Furthermore, the thixotropic index (TI), calculated as the ratio of the viscosity at 0.2 rpm and 2 rpm, ranges from 1.2 to 5.2. Typical commercial potting compounds require a viscosity between 1000 and 120,000 cPs at 2 rpm and a TI of 1.0–2.0 to ensure processability.

While the addition of benzoxazine (BZ) to an epoxy resin effectively reduces the coefficient of thermal expansion (CTE), the unfilled 60/40 epoxy/BZ mixture still possesses high CTE values (*α*_1_ = 95 ppm/°C, *α*_2_ = 162 ppm/°C) that are substantially larger than those of the microelectronic components they are designed to protect. The CTE of a standard silicon die typically ranges from 2.6 to 3.3 ppm/°C, which is roughly 30 to 50 times lower than that of the neat 60/40 epoxy/BZ blend [21]. Consequently, a high filler loading is required to minimize the CTE mismatch while maintaining processability. Table 2 presents the CTE values below (*α*_1_, 0–100 °C) and above (*α*_2_, 190–250 °C) the glass transition temperature (*T*_g_) for various bimodal and trimodal composites. Irrespective of the filler type, the CTE consistently decreases as the filler content increases. In particular, the bimodal composites with BN exhibit the lowest CTE values at an equivalent filler loading. This reduction is driven by the inherently lower CTE of ATH or ceramic particles compared to the neat resin. Furthermore, in the trimodal composites with a fixed total filler loading of 40 wt%, the CTE exhibits a slight decrease as the ratio of BN to Al_2_O_3_ increases.

**Figure 1:**
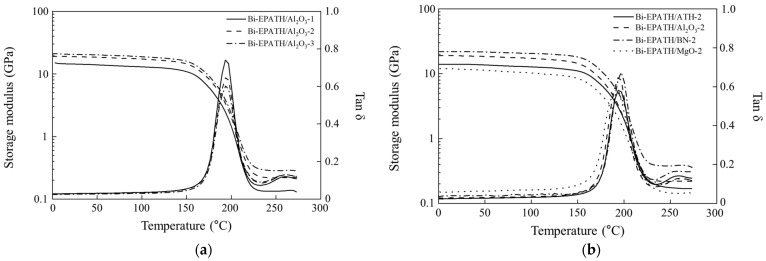
Storage modulus ($E^{\prime }$) and loss factor (tan δ) curves of (**a**) bimodal composites containing different amounts of Al_2_O_3_ and (**b**) different ceramic fillers with the same content.

Figure 1 shows the plots of storage modulus ($E^{\prime }$) and loss factor (tan δ) curves of bimodal composites. The neat epoxy/benzoxazine mixture produced thin and brittle films that are unsuitable for mechanical characterization. The storage modulus of epoxy/BZ composite containing 30 wt% EPATH was approximately 8 GPa at 25 °C. As shown in Figure 1a, the storage modulus increases to 14 GPa at 25 °C with the addition of 20 wt% Al_2_O_3_ and reaches 18 GPa 30 wt%. At a constant loading of 30 wt%, the storage modulus of the composites decreases in the following order: BN > Al_2_O_3_ > ATH > MgO (Figure 1b). The high modulus of the BN composites is attributed to the large volume occupied by the BN agglomerate. A vast polymer–filler interphase severely restricts polymer chain mobility and thereby stiffens the matrix. The addition of ATH or ceramics shifts the tan δ peak to a higher temperature due to the restricted mobility of the polymer chains. While these fillers elevate the *T*_g_ by approximately 5 °C relative to the base composite containing 30 wt% EPATH, the *T*_g_ remains largely invariant across filler loadings ranging from 20 to 40 wt%. Additionally, the amplitude of the tan δ peak is suppressed (Figure S2). The addition of inherently rigid ceramic fillers reduces the free spaces between epoxy/benzoxazine macromolecules and restricts molecular chain rotation, thereby decreasing the overall energy dissipation capability of the composite system. The crosslinking density increases to 0.009, 0.016, 0.024 mol/cm^3^ at Al_2_O_3_ loadings of 20, 30, and 40 wt%, respectively. The relative stability of the *T*_g_ within 2~3 °C suggests that the calculated density is predominantly influenced by the filler-induced increase in the rubbery plateau. This rise suggests that the rigid ceramic fillers effectively restrict the mobility of epoxy/benzoxazine chains. At a constant filler loading of 30 wt%, the crosslinking density varied depending on the filler type. Boron nitride (BN) exhibits the highest density of 0.029 mol/cm^3^, whereas magnesium oxide (MgO) shows the lowest value of 0.012 mol/cm^3^.

**Figure 2:**
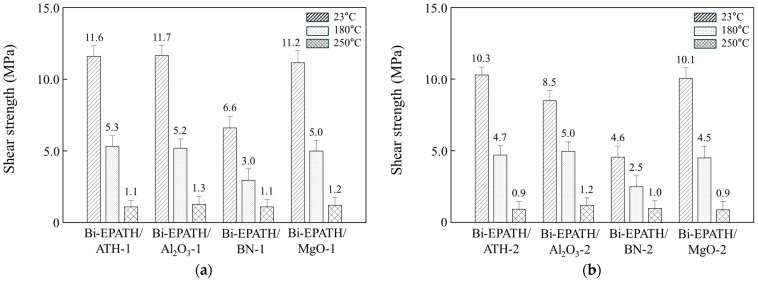
Die shear strength of various bimodal composites with filler contents of (**a**) 20 wt% and (**b**) 30 wt%. The measurement was conducted between a silicon die and a silver-plated lead frame.

Figure 2 illustrates the die shear strength of the bimodal composites containing different ceramic fillers at 20 wt% and 30 wt% loading. The adhesion between a silicon (Si) die and a silver (Ag)-plated lead frame was assessed not only at 23 °C but also at 180 °C and 250 °C, which correspond to typical wire bonding and solder reflow temperatures, respectively. An inverse relationship between filler content and shear strength is generally observed (Figure S3). At an equivalent loading, the die shear strength is highly dependent on the type of ceramic filler. Furthermore, the shear strength undergoes a significant reduction at elevated temperatures. The behavior is attributed to the combined effects of the polymer matrix softening induced by the transition to a rubbery state near *T*_g_ and the thermal stresses generated by the coefficient of thermal expansion (CTE) mismatch with the Si die (2.6 ppm/°C) and the Ag lead frame (19.1 ppm/°C). As confirmed in Figure 1 and Table 1, the composites exhibit a *T*_g_ between 192 and 200 °C, with the CTE surpassing 100 ppm/°C above *T*_g_ depending on the composition. At 250 °C, all epoxy/benzoxazine composites are well above their *T*_g_, residing entirely within the rubbery plateau region, as observed in Figure 1. The Bi-EPATH/ATH (20 wt% ATH) exhibits die shear strength of 11.6 MPa at 23 °C, 5.3 MPa at 180 °C, and 1.1 MPa at 250 °C (Figure 2a). A similar trend is observed upon increasing the ceramic filler loading to 30 wt% (Figure 2b). Notably, the BN-filled composites display significantly lower shear strength at the same loading. At 20 wt% and 30 wt% loadings, the strength of the BN composites reached only about 56% and 54% of the Al_2_O_3_ equivalents at 23 °C, respectively. This is primarily because the low bulk density of BN causes it to occupy a much larger volume fraction at the same weight percentage. Furthermore, the chemically inert surface of BN restricts its interaction with the resin matrix, which generates interfacial defects and undermines the macroscopic shear resistance.

**Figure 3:**
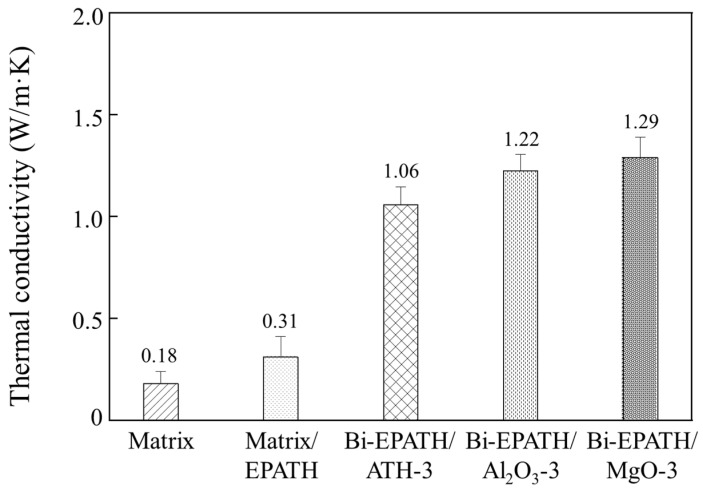
Thermal conductivity of neat epoxy/BZ rein, a composite with 30 wt% EPATH, and bimodal composites consisting of 30 wt% EPATH and 40 wt% ceramic fillers.

Figure 3 presents a comparison of mean thermal conductivity and the corresponding standard deviation of the neat epoxy/BZ matrix, the 30 wt% EPATH composite, and various bimodal composites with a total filler loading of 70 wt% obtained via the addition of 40 wt% ATH or ceramic fillers. Results for the bimodal BN system are not presented due to processing limitations at loading above 30 wt%. It is widely recognized that the BN-filled composites display anisotropic thermal conductivity owing to the preferential in-plane alignment [23,24,25]. The neat epoxy/BZ matrix itself exhibits a low thermal conductivity of 0.18 W/m∙K. Upon the addition of 30 wt% EPATH, the thermal conductivity slightly increases to 0.31 W/m∙K, which remains insufficient for demanding thermal management applications. The bimodal composites exhibit thermal conductivity above 1.0 W/m∙K at 40 wt% regardless of the secondary filler type. Specifically, the incorporation of MgO and Al_2_O_3_ yields higher values of 1.29 W/m∙K and 1.22 W/m∙K, compared to the ATH-filled composites (1.05 W/m∙K). Given the similarities in mean particle sizes and spherical shape, the disparity is directly ascribed to the superior intrinsic thermal conductivities of the ceramic particles.

### 3.2. Thermal and Mechanical Properties of Trimodal Epoxy/Benzoxazine Composites

**Figure 4:**
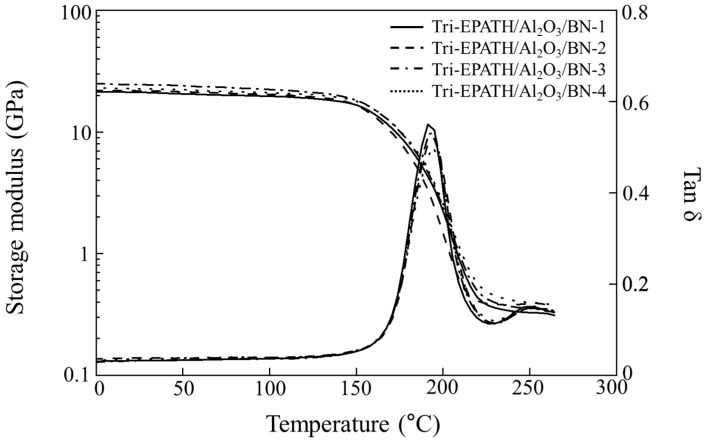
Storage modulus $\left(E^{\prime }\right)$ and tan δ curves of various trimodal composites with varying Al_2_O_3_ to BN ratios at a fixed total filler loading of 40 wt%.

Considering overall properties of bimodal composites along with cost-effectiveness, Al_2_O_3_ was chosen as the primary filler. Trimodal composites were subsequently prepared by progressively substituting Al_2_O_3_ with BN in 2.5 wt% increments, while maintaining a constant total filler loading of 70 wt%. Figure 4 illustrates the storage modulus ($E^{\prime }$) and tan δ profiles of the trimodal composites as a function of the Al_2_O_3_/BN ratios. As the BN content increases from 2.5 wt% (Tri-EPATH/Al_2_O_3_/BN-1) to 7.5 wt% (Tri-EPATH/Al_2_O_3_/BN-3), the storage modulus rises from 21.8 GPa to 24.5 GPa, while the peak intensity of the tan δ curve is systematically suppressed. The storage modulus of the bimodal composite with 40 wt% Al_2_O_3_ is 20.5 GPa at 25 °C. Although the weight percentage of the trimodal composites remains constant, the higher volume fraction of BN imposes greater confinement on the polymer matrix, limiting molecular motion. Irrespective of the Al_2_O_3_/BN ratio, all trimodal composites exhibit a *T*_g_ in the range of 193–195 °C. These restricted chains fail to dissipate applied stress efficiently, leading to the observed reduction in the tan δ peak. When BN content is further increased to 10 wt%, the storage modulus decreases to below 22.7 GPa. This deterioration is attributed to BN particle agglomeration, which initiates defect formation rather than providing mechanical reinforcement.

**Figure 5:**
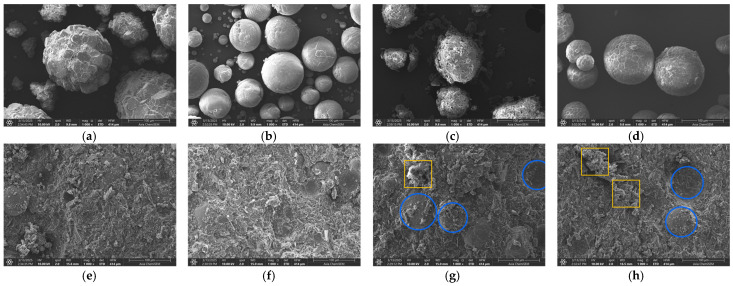
SEM images of pure particles of (**a**) ATH, (**b**) Al_2_O_3_, (**c**) BN, (**d**) MgO, and the fracture surface of trimodal composites with various Al_2_O_3_/BN ratio of (**e**) Tri-EPATH/Al_2_O_3_/BN-1, (**f**) Tri-EPATH/Al_2_O_3_/BN-2, (**g**) Tri-EPATH/Al_2_O_3_/BN-3, and (**h**) Tri-EPATH/Al_2_O_3_/BN-4.

Figure 5 presents scanning electron microscopy (SEM) micrographs of the pristine ATH, Al_2_O_3_, BN, and MgO particles (a–d), and the fracture surfaces of the trimodal composites containing Al_2_O_3_ and BN (e–h). Although the macroscopic shape of the particles is spherical, their size distributions are non-uniform. While the Al_2_O_3_ and MgO powders form dense and monolithic clusters, the ATH and BN powders manifest as agglomerations of plate-like subunits. Especially, the constituent subunits of BN are more loosely bounded, making their boundaries identifiable (Figure 5c). A cross-sectional analysis of the trimodal composites reveals that Al_2_O_3_ (denoted by blue circles) retains its original spherical shape within the matrix, whereas the BN agglomerates are no longer identifiable, presumably due to their fragmentation of agglomerates during the compounding processing. In certain regions, cavities resulting from the pull-out of spherical particles can be observed. At BN loadings of 5 wt% BN or less, the composites exhibit a relatively smooth surface with the filler particles homogeneously dispersed throughout the polymer matrix (Figure 5e,f). However, as the BN loading increases to 7.5 wt%, the agglomeration of irregular fragments (denoted by orange squares) occurs in localized regions, resulting in a rougher surface. This agglomeration phenomenon becomes even more pronounced when the BN content is further increased to 10 wt% (Figure 5g,h).

**Figure 6:**
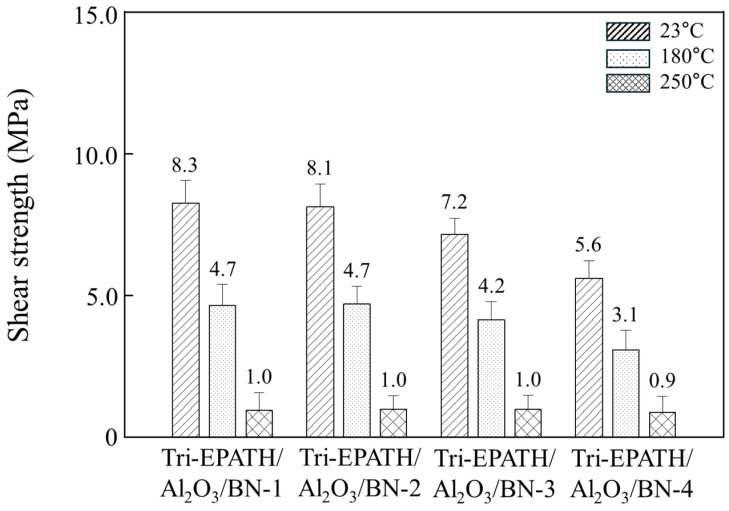
Die shear strength of various trimodal composites with varying Al_2_O_3_ to BN ratios at a fixed total filler loading of 40 wt%. The measurement was conducted between a silicon die and a silver-plated lead frame.

Figure 6 illustrates the temperature-dependent shear strength of the trimodal composites. Consistent with the behavior of the bimodal systems, the shear strength undergoes a significant reduction when the test temperature is elevated to 180 °C and 250 °C. With low BN content of 5 wt% or less, i.e., Tri-EPATH/Al_2_O_3_/BN-1 and Tri-EPATH/Al_2_O_3_/BN-2, no obvious differences in shear strength are observed across the entire temperature range. For comparison, it should be noted that the shear strength of the bimodal composites containing Al_2_O_3_ and MgO at an equivalent total composition was 5.3 MPa and 4.8 MPa, respectively. When the BN content is increased to 7.5 wt% (Tri-EPATH/Al_2_O_3_/BN-3), the shear strength decreases to 7.2 MPa at 23 °C and 4. 2 MPa at 180 °C. At a higher loading of 10 wt% BN (Tri-EPATH/Al_2_O_3_/BN-4), the shear strength undergoes further reduction of 32% at 23 °C, 31% at 180 °C, and 10% at 250 °C, respectively, relative to the 2.5 wt% BN composites. As observed in the SEM analysis, the presence of the BN agglomerates at loadings above 7.5 wt% exerts a distinctly detrimental effect on the adhesion, causing premature mechanical failure under relatively low stress. Furthermore, the high viscosity exceeding 602,700 cPs, coupled with the chemically inert nature, hinders uniform wetting on the Ag substrate.

**Figure 7:**
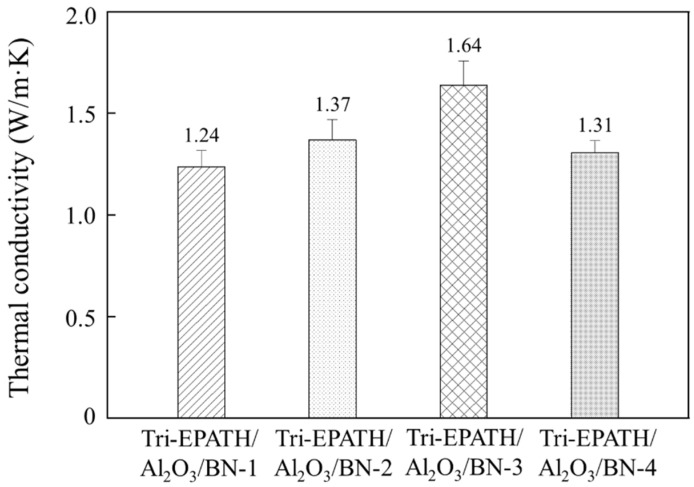
Thermal conductivity of various trimodal composites with varying Al_2_O_3_ to BN ratios at a fixed total filler loading of 40 wt%.

The effect of the Al_2_O_3_ to BN ratio on the thermal conductivity of the trimodal composites is examined. As shown in Figure 7, while the bimodal composite containing 40 wt% Al_2_O_3_ exhibits a thermal conductivity of 1.22 W/m∙K, a steady enhancement is observed upon the progressive substitution of Al_2_O_3_ with BN. The thermal conductivity reaches a maximum value of 1.64 W/m∙K at 7.5 wt% BN. Microscopically, individual plate-like BN particles formed from the fragmentation of larger agglomerates during processing are dispersed uniformly within the matrix. These platelets connect the neighboring micro-spherical Al_2_O_3_ particles that constitute the primary thermally conductive network, thereby constructing highly efficient phonon transmission pathways. The synergistic combination of micro-sized spherical Al_2_O_3_ and plate-like BN optimizes the packing density and generates additional thermal conduction channels. However, at a higher BN loading of 10 wt%, the thermal conductivity drops to 1.31 W/m∙K. This reduction is attributed to the re-agglomeration of BN particles at high concentrations.

### 3.3. Water Absorption and Shear Strength of Trimodal Composites

**Figure 8:**
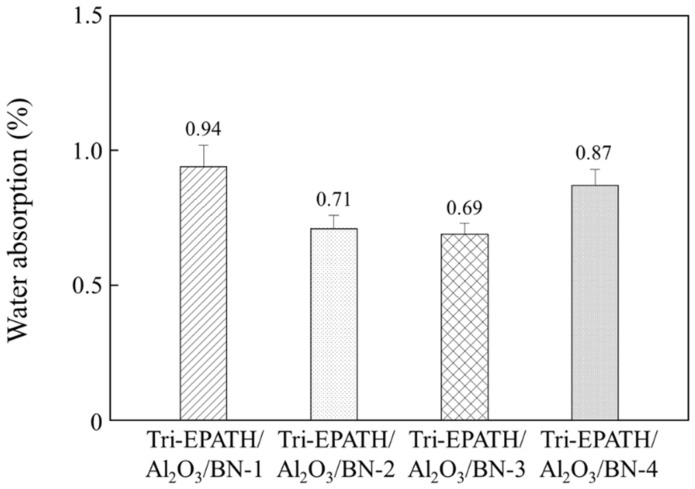
Water absorption of trimodal composites with varying Al_2_O_3_ to BN ratios at a fixed total filler loading of 40 wt%. The value was measured after immersion at room temperature for 24 h.

The presence of absorbed moisture within microelectronic packages can induce softening of the encapsulant materials and delamination driven by vaporization during reflow soldering. Although blending hydrophilic epoxy with benzoxazine mitigates the water absorption, the filler plays a dominant role at high loading. By increasing the filler content, the volume of the moisture-vulnerable organic resin matrix is reduced. Water absorption profiles of the trimodal composites are plotted in Figure 8. As the BN content increases from 2.5 wt% (Tri-EPATH/Al_2_O_3_/BN-1) to 7.5 wt% Tri-EPATH/Al_2_O_3_/BN-3), the total water absorption systematically decreases from 0.94% to 0.69%. For comparison, the bimodal composites containing 40 wt% Al_2_O_3_ and MgO exhibit higher absorption values of 1.62% and 1.19%, respectively. The high water absorption of Al_2_O_3_ originates from its free –OH groups on the surface, which chemically absorb water via hydrogen bonding, and physical adsorption to form Al_2_O_3_∙H_2_O [26,27]. However, BN is characterized by a chemically inert surface that is devoid of –OH groups and difficult to wet with polymer resins [28,29]. This hydrophobic nature effectively repels moisture, leading to superior moisture resistance. Therefore, by increasing the BN content, the volume of the water-vulnerable Al_2_O_3_ and organic resin is reduced.

**Figure 9:**
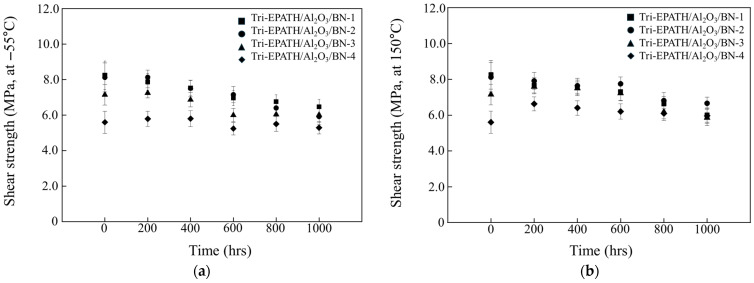
Variation of die shear strength of trimodal composites according to continuous exposure for 1000 h at (**a**) −55 °C and (**b**) 150 °C.

The low- and high-temperature endurability of the trimodal composites has been evaluated by sequential exposure at −55 °C and 150 °C, respectively. As shown in Figure 9, the time-dependent behavior of shear strength differs depending on the composition. When aged at −55 °C, although the rate of decline varied across time intervals, the shear strength generally exhibited a downward trend. Following 1000 h of exposure, the average shear strength of Tri-EPATH/Al_2_O_3_/BN-1, Tri-EPATH/Al_2_O_3_/BN-2, Tri-EPATH/Al_2_O_3_/BN-3, and Tri-EPATH/Al_2_O_3_/BN-4 shows reductions of approximately 22.0%, 26.9%, 16.4%, and 5.5%, respectively, compared to their initial values before aging. Upon exposure to 150 °C, the behavior is dependent on their composition. While Tri-EPATH/Al_2_O_3_/BN-1 and Tri-EPATH/Al_2_O_3_/BN-2 show an overall decrease in average shear strength over time, the other specimens, i.e., Tri-EPATH/Al_2_O_3_/BN-3 and Tri-EPATH/Al_2_O_3_/BN-4, display an initial increase followed by a subsequent decline. After 1000 h, the average shear strength of Tri-EPATH/Al_2_O_3_/BN-1 to Tri-EPATH/Al_2_O_3_/BN-3 decreases by 27.7%, 17.8%, and 18.6%, respectively, whereas Tri-EPATH/Al_2_O_3_/BN-4 exhibits an increase of 6.4%. Tri-EPATH/Al_2_O_3_/BN-4 exhibits consistently high shear strength throughout the aging process, showing increases of 18.4%, 14.3%, 10.7%, and 8.9% after 200, 400, 600, and 800 h, respectively. This behavior is attributed to the additional crosslinking of the unreacted epoxy/benzoxazine component during continuous exposure to high temperature. According to DSC thermograms of the epoxy/benzoxazine mixture, the onset of the reaction occurs around 130 °C.

## 4. Conclusions

This study investigated the simultaneous enhancement of flame retardancy and heat dissipation in bimodal and trimodal composites by incorporating Al_2_O_3_, MgO, or BN fillers into a UL94 V-0 epoxy/benzoxazine/EPATH (30 wt%) composite. The addition of the ceramic fillers improved both the storage modulus and thermal conductivity. The composites with 40 wt% Al_2_O_3_ or MgO achieved thermal conductivities of 1.22 W/m∙K and 1.29 W/m∙K, respectively. The incorporation of BN was constrained above 30 wt% because its relatively large volume at the same weight fraction led to a substantial rise in viscosity, which limited its further incorporation. Furthermore, the chemical inertness resulted in superior water absorption and inferior shear strength compared to Al_2_O_3_ and MgO composites. The trimodal composites incorporating both Al_2_O_3_ and BN exhibited an enhanced thermal conductivity exceeding 1.6 W/m∙K, where the BN platelets connect the neighboring micro-spherical Al_2_O_3_ particles, thereby constructing highly efficient phonon transmission pathways. However, at BN loadings above 7.5 wt%, the formation of particle agglomerates adversely affected the composite by reducing thermal conductivity and promoting water absorption. The trimodal composite showed a shear strength reduction of less than 30% even after 1000 h of exposure at −55 °C and 150 °C. These thermal conductive and flame-retardant composites are expected to play a critical role in ensuring the safety and reliability of encapsulants for high-power, highly integrated electronic devices.

## Figures and Tables

**Table 1:** Composition of bimodal and trimodal epoxy/benzoxazine composites containing epoxy-silane-treated ATH (EPATH) and ceramic fillers.

Composition	Resin	Flame Retardant		Ceramic Fillers		
	Epoxy/BZ	EPATH	ATH	Al_2_O_3_	BN	MgO
Bi-EPATH/ATH-1	50	30	20			
Bi-EPATH/ATH-2	40	30	30			
Bi-EPATH/ATH-3	30	30	40			
Bi-EPATH/Al_2_O_3_-1	50	30		20	/	/
Bi-EPATH/Al_2_O_3_-2	40	30		30	/	/
Bi-EPATH/Al_2_O_3_-3	30	30		40	/	/
Bi-EPATH/BN-1	50	30		/	20	/
Bi-EPATH/BN-2	40	30		/	30	/
Bi-EPATH/MgO-1	50	30		/	/	20
Bi-EPATH/ MgO-2	40	30		/	/	30
Bi-EPATH/ MgO-3	30	30		/	/	40
Tri-EPATH/Al_2_O_3_/BN-1	30	30		37.5	2.5	/
Tri-EPATH/Al_2_O_3_/BN-2	30	30		35	5	/
Tri-EPATH/Al_2_O_3_/BN-3	30	30		32.5	7.5	/
Tri-EPATH/Al_2_O_3_/BN-4	30	30		30	10	/

**Table 2:** Composition-dependent thermal and mechanical properties of epoxy/benzoxazine mixtures.

Composition	Vicosity (cPs)			CTE (ppm/°C)	
	0.2 rpm	2 rpm	TI	*α*_1_ (0–100 °C)	*α*_2_ (190–250 °C)
Bi-EPATH/ATH-1	74,560	14,290	5.2	30.1	126
Bi-EPATH/ATH-2	103,600	34,380	3.0	26.5	112
Bi-EPATH/ATH-3	194,700	119,300	1.6	25.6	108
Bi-EPATH/Al_2_O_3_-1	35,210	21,750	1.6	33.5	127
Bi-EPATH/Al_2_O_3_-2	107,700	69,800	1.5	27.2	117
Bi-EPATH/Al_2_O_3_-3	138,800	90,100	1.5	20.7	103
Bi-EPATH/BN-1	372,800	167,600	2.2	24.5	125
Bi-EPATH/BN-2	Not Available	Not Available	Not Available.	21.4	118
Bi-EPATH/MgO-1	82,850	16,570	5.0	28.7	131
Bi-EPATH/ MgO-2	93,120	21,130	4.4	26.1	121
Bi-EPATH/ MgO-3	111,800	95,480	1.2	21.1	107
Tri-EPATH/Al_2_O_3_/BN-1	138,800	99,420	1.4	26.0	109
Tri-EPATH/Al_2_O_3_/BN-2	281,700	154,900	1.8	25.5	104
Tri-EPATH/Al_2_O_3_/BN-3	602,700	Not Available	Not Available	26.4	104
Tri-EPATH/Al_2_O_3_/BN-4	1,121,000	Not Available.	Not Available.	28.8	105

## Data Availability

The original contributions presented in this study are included in the article/Supplementary Materials. Further inquiries can be directed to the corresponding author.

## Supplementary Materials

The following supporting information can be downloaded at: https://www.mdpi.com/article/10.3390/polym18050648/s1, Figure S1: Isothermal DSC thermograms of epoxy/benzoxazine mixture, epoxy/benzoxazine with 30 wt% EPATH, and epoxy/benzoxazine/EPATH with 40 wt% Al_2_O_3_ composites measured at 175 °C.; Figure S2: Storage modulus ($E^{\prime }$) and loss factor (tan δ) curves of bimodal composites containing different amount of (a) ATH and (b) MgO.; Figure S3: Die shear strength of bimodal composites containing different amount of (a) ATH, (b) Al_2_O_3_, and (c) MgO.
