# Supplementary material for: Tailoring Flame Retardance and Thermal Conductivity of Epoxy/Benzoxazine Mixtures via Aluminum Trihydrate and Ceramic Hybridization
Source: Polymers (Basel). 2026 Mar 6;18(5):648. doi: 10.3390/polym18050648 (PMC12987129; doi:10.3390/polym18050648)
Supplement: Supplementary file 1 [file polymers-18-00648-s001.zip › polymers-4180108-supplementary.pdf]

Tailoring Flame Retardance and Thermal Performances of Epoxy/Benzoxazine via Aluminum Trihydrate and Ceramic Hybridization

Kyung-Soo Sung[1,2], Hye-Won Cho[2], Kyu-Hwan Kwon[1], and Namil Kim[1]*

[1] *Department of Chemical Engineering, Hannam University, Daejeon 34054, Korea*
[2] *Korea Research & Development Center, Protavic Korea, Daejeon 34326, Korea*

*Corresponding author email: nikim@hnu.kr

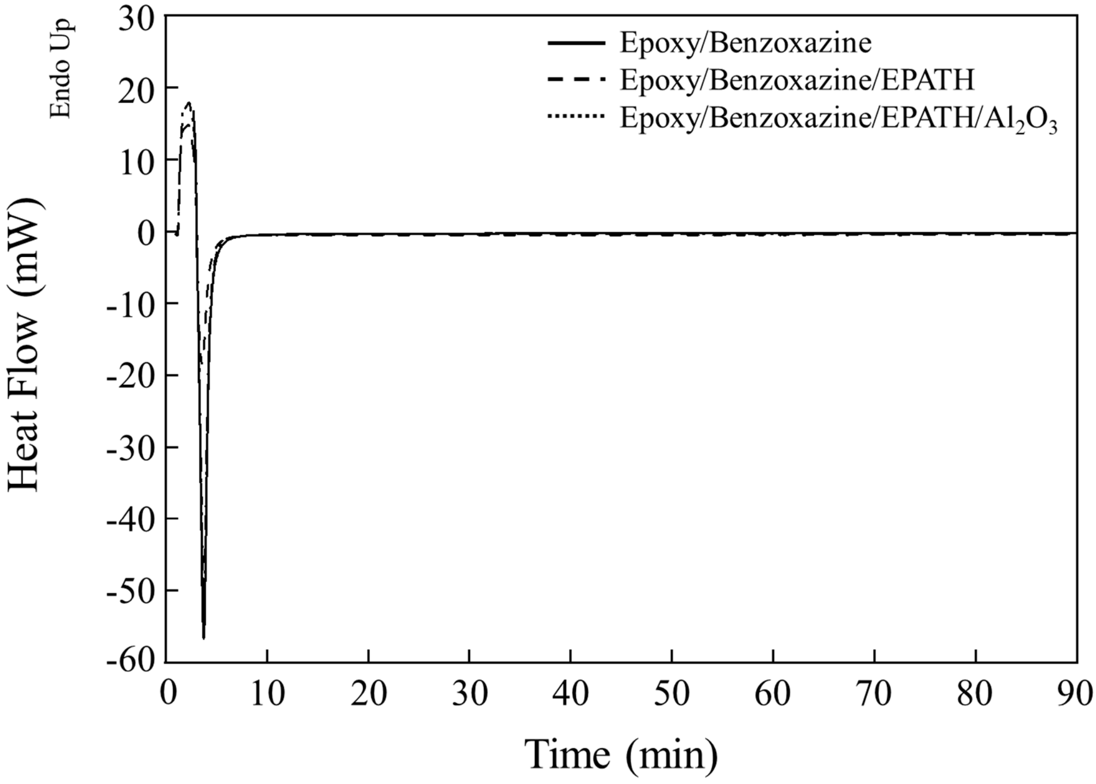

Figure S1. Isothermal DSC thermograms of epoxy/benzoxazine mixture, epoxy/benzoxazine with 30 wt% EPATH, and epoxy/benzoxazine/EPATH with 40 wt% Al_2O_3 composites measured at 175 ^oC.

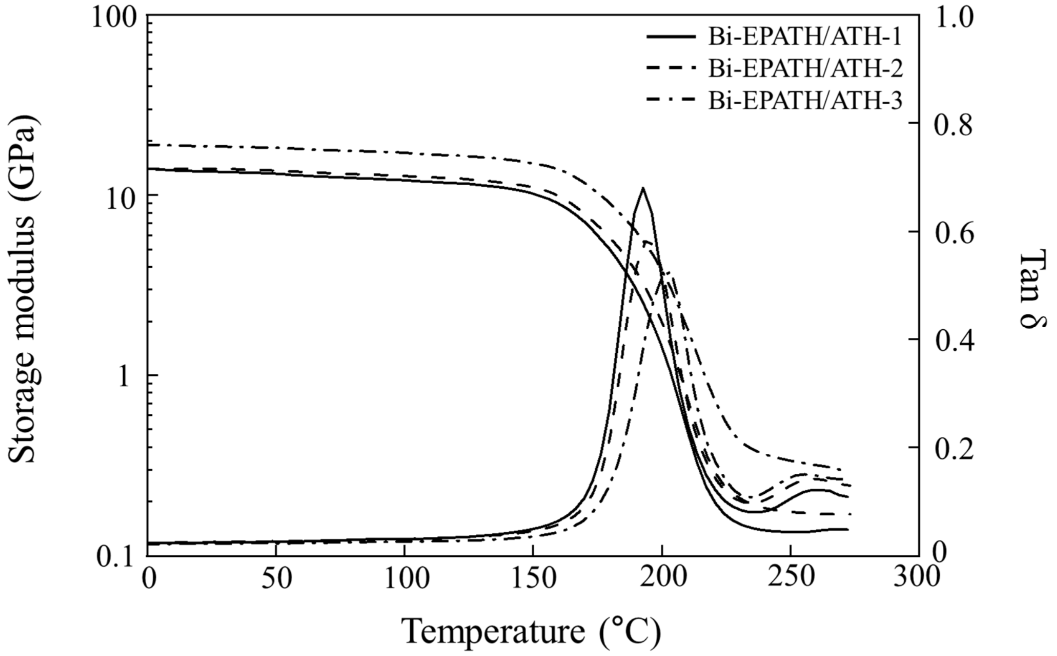

(a)

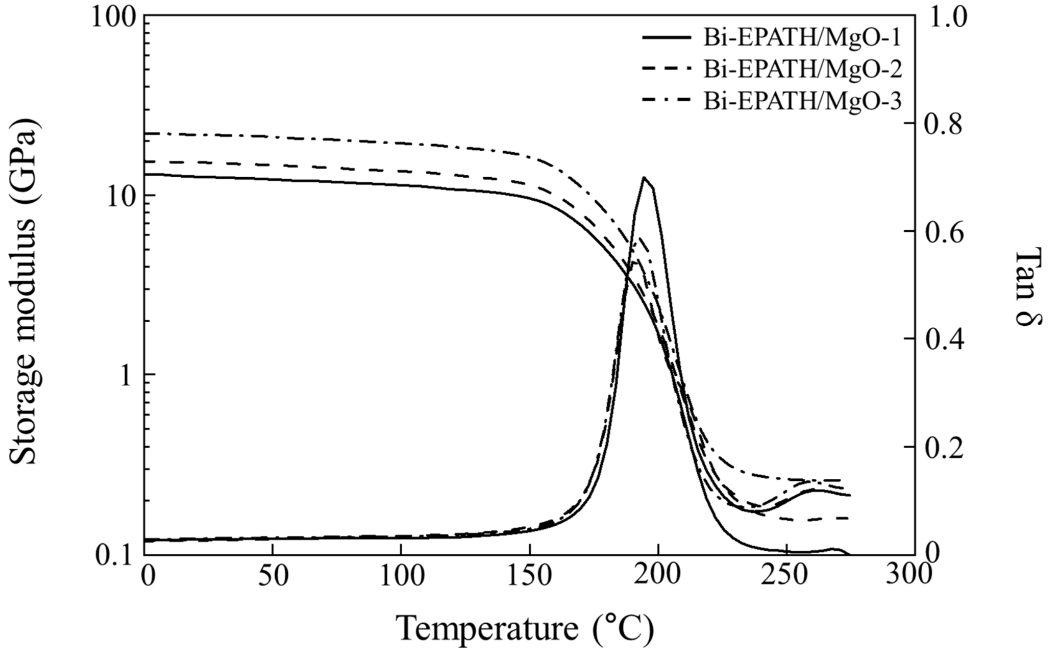

(b)

Figure S2. Storage modulus (E') and loss factor (tan δ) curves of bimodal composites containing different amount of (a) ATH and (b) MgO.

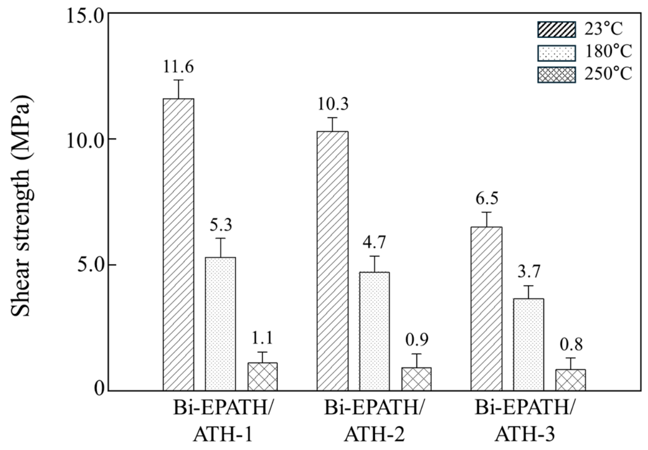

(a)

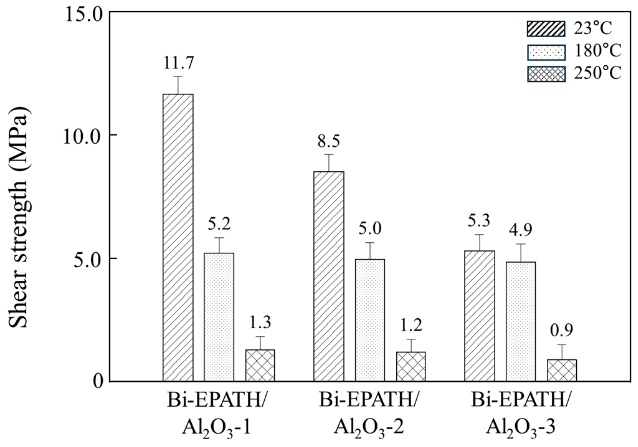

(b)

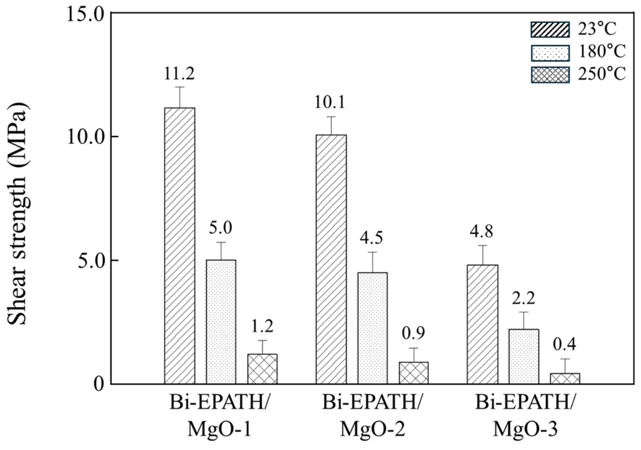

(c)

Figure S3. Die shear strength of bimodal composites containing different amount of (a) ATH, (b) Al_2O_3, and (c) MgO.
